# Supplementary figures and images for: Construction and Experimental Validation of a Petri Net Model of Wnt/β-Catenin Signaling
Source: PLoS One. 2016 May 24;11(5):e0155743. doi: 10.1371/journal.pone.0155743 (PMC4878796; doi:10.1371/journal.pone.0155743)

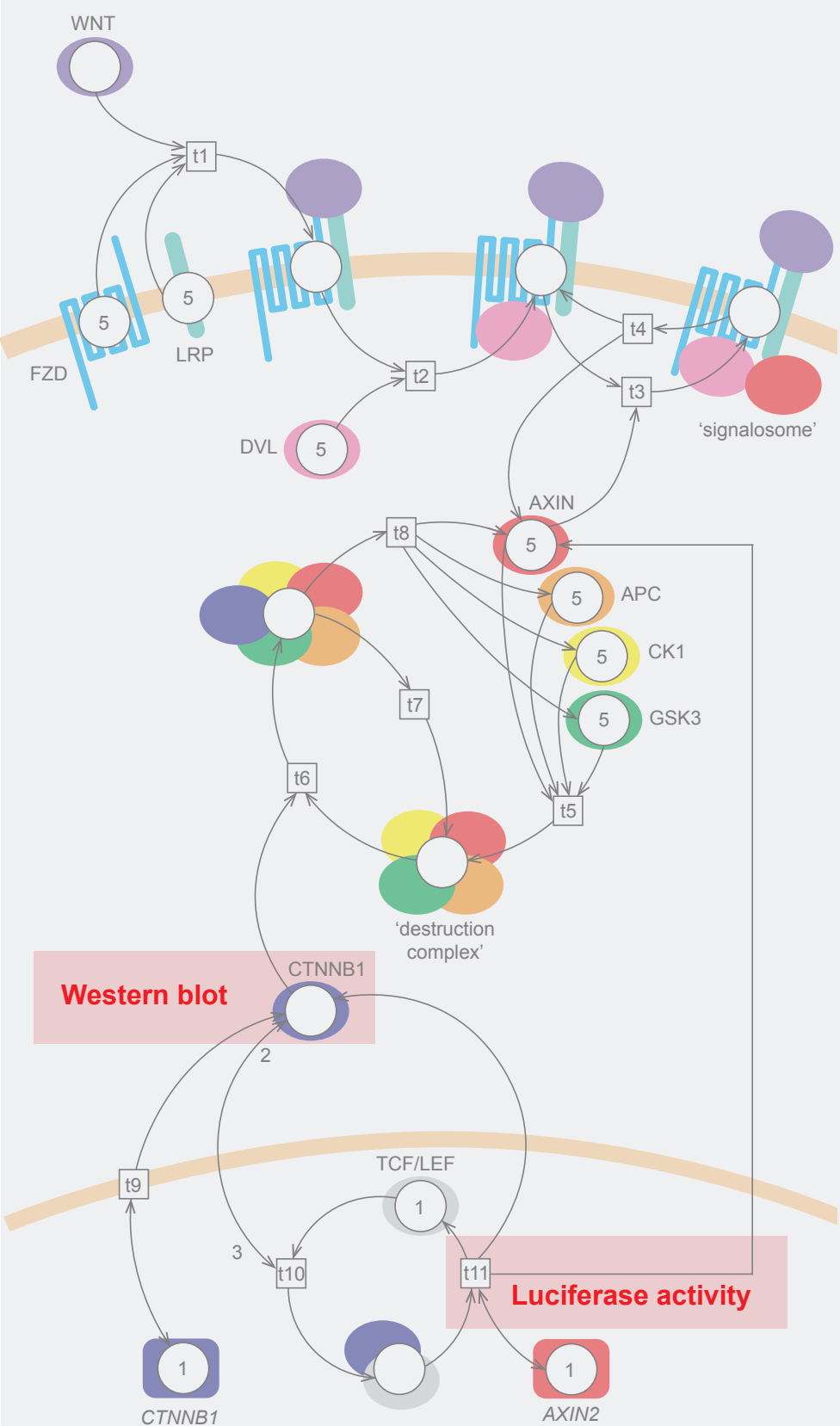

Supplement: S1 Fig — The “location” of the readouts of Western blot analysis and luciferase reporter assay compared to their equivalent implementation in our Petri net model. Western blot analysis measures the protein levels of β-catenin (i.e. measures the β-catenin accumulation), whereas the luciferase reporter assay measures the transcriptional activation (by β-catenin/TCF complexes) of the TOPFLASH reporter, which is followed by the production of luciferase protein. Since the luciferase reporter activation requires β-catenin to be present it takes longer for the TOPFLASH reporter to be activated than for the increase in β-catenin protein levels to occur i.e. the activation always occurs downstream of β-catenin accumulation. (PDF) [file pone.0155743.s001.pdf]
